# Abundant local interactions in the 4p16.1 region suggest functional mechanisms underlying *SLC2A9* associations with human serum uric acid

**DOI:** 10.1093/hmg/ddu227

**Published:** 2014-05-12

**Authors:** Wen-Hua Wei, Yunfei Guo, Alida S.D. Kindt, Tony R. Merriman, Colin A. Semple, Kai Wang, Chris S. Haley

**Affiliations:** 1 MRC Human Genetics Unit, Institute of Genetics and Molecular Medicine, University of Edinburgh, Western General Hospital, Crewe Road, Edinburgh EH4 2XU, UK,; 2 Arthritis Research UK Centre for Genetics and Genomics, Institute of Inflammation and Repair, Faculty of Medical and Human Sciences, Manchester Academic Health Science Centre, University of Manchester, Oxford Road, Manchester M13 9PT, UK,; 3 Zilkha Neurogenetic Institute, University of Southern California, Los Angeles, CA, USA; 4 Department of Biochemistry, University of Otago, PO Box 56, Dunedin, New Zealand

## Abstract

Human serum uric acid concentration (SUA) is a complex trait. A recent meta-analysis of multiple genome-wide association studies (GWAS) identified 28 loci associated with SUA jointly explaining only 7.7% of the SUA variance, with 3.4% explained by two major loci (*SLC2A9* and *ABCG2*). Here we examined whether gene–gene interactions had any roles in regulating SUA using two large GWAS cohorts included in the meta-analysis [the Atherosclerosis Risk in Communities study cohort (ARIC) and the Framingham Heart Study cohort (FHS)]. We found abundant genome-wide significant local interactions in ARIC in the 4p16.1 region located mostly in an intergenic area near *SLC2A9* that were not driven by linkage disequilibrium and were replicated in FHS. Taking the forward selection approach, we constructed a model of five SNPs with marginal effects and three epistatic SNP pairs in ARIC—three marginal SNPs were located within *SLC2A9* and the remaining SNPs were all located in the nearby intergenic area. The full model explained 1.5% more SUA variance than that explained by the lead SNP alone, but only 0.3% was contributed by the marginal and epistatic effects of the SNPs in the intergenic area. Functional analysis revealed strong evidence that the epistatically interacting SNPs in the intergenic area were unusually enriched at enhancers active in ENCODE hepatic (HepG2, *P* = 4.7E−05) and precursor red blood (K562, *P* = 5.0E−06) cells, putatively regulating transcription of *WDR1* and *SLC2A9*. These results suggest that exploring epistatic interactions is valuable in uncovering the complex functional mechanisms underlying the 4p16.1 region.

## INTRODUCTION

Human serum uric acid concentration (SUA) is the outcome of balancing production (primarily in the liver) against excretion (mostly in the kidney) (1). High SUA (i.e. hyperuricaemia) can lead to gout (2). SUA is a complex trait with estimated heritability ranging from 40 to 70% (3–5). A recent meta-analysis comprising >140 000 individuals of European ancestry identified 18 novel loci in addition to 10 previous known that jointly explained only 7.7% of the SUA variance, of which 3.4% was explained by *SLC2A9* and *ABCG2* (6). The meta-analysis results reiterate the ‘missing heritability’ issue (7,8) but reinforce the impression that increasing the sample size is effective in discovering novel loci but with decreasing effects (9–11). Gene–gene interactions (epistasis)—a potential source of SUA variation, were not considered in the meta-analysis study (6). Tools for analysing epistasis at the genome-wide level currently can only handle SNPs with precise genotypes (12–16) and thus are unable to support meta-analysis of epistasis that requires imputed SNPs with probability-attached genotypes.

In contrast to the great success in genome-wide association studies (GWAS) (attributable mostly to meta-analysis) (9), the genome-wide search for epistasis in individual GWAS populations so far has been disappointing in general (17,18). This may not be too surprising because the power of detection of pairwise epistasis is a function of the interaction effect and sample size as well as linkage disequilibrium (LD) between a genotyped SNP and underlying causal variants at both loci (rather than one locus in conventional GWAS). Overall one requires a much larger sample size (18,19) than offered in each individual GWAS population. The low power issue is amplified by the need to apply significance thresholds derived from Bonferroni correction of billions of multiple tests with consensus thresholds (like 5.0E−08 for GWAS) not yet available (20). The high-density SNP coverage of the genome that is essential to provide sufficient LD for detecting epistasis is not available in most GWAS cohorts genotyped with older, relatively low-density SNP chips (21–24), posing difficulties to both detection and replication of epistatic signals. For example, in our previous study of epistasis in SUA using small isolated populations genotyped by chips with ∼300 000 SNPs, interactions involving *SLC2A9* were detected but could not be robustly replicated (25).

At least two additional approaches could potentially increase power of detection of epistasis in single populations. First, to detect interactions involving SNPs with important marginal effects (marginal SNPs) based on a specific significance threshold adjusted for a much reduced number of tests (14,21,26–29). Second, to examine local interactions between neighbouring SNPs in low LD, e.g. two SNPs located within 1 Mb on the same chromosome and with an interaction *P*-value (*P*_int_) of <1.0E−05 (21,24). Such local interactions may exist within a gene or between neighbouring genes (30,31), and rather than capturing functional genetic interactions could potentially capture variants missing from GWAS via haplotype effects (24,32) and provide new insights into the underlying molecular mechanisms (33,34). Both approaches require no prior biological knowledge (23) and thus can provide a useful view of interactions complementary to conventional GWAS (24).

Here we used two large cohorts included in the GWAS meta-analysis (6), i.e. the Atherosclerosis Risk in Communities study cohort (ARIC) and the Framingham Heart Study cohort (FHS) both genotyped with >500 000 SNPs, to re-examine epistasis in SUA comprehensively. We used the ARIC samples with European ancestry for discovery and the FHS cohort (excluding samples in generation one) for replication. We performed full pairwise genome scans for both cohorts using a fast tool BiForce (12) and examined SNP interactions in three categories—with and without marginal SNPs and local interactions, and using specific significance thresholds derived following the procedures previously defined (21,24).

## RESULTS

After careful data scrutiny and quality control (see Materials and Methods), 514 662 SNPs and 9172 samples (4884 females) in ARIC and 410 947 SNPs and 5538 samples (2951 females) in FHS were used in subsequent data analyses (Supplementary Material, Table S1). SNP positions quoted in this study are based on the human genome build (UCSC hg19/NCBI 37.3). Conventional GWAS identified 166 genome-wide significant (*P* < 5.0E−08) SNP associations in ARIC (Supplementary Material, Table S2 and Fig. S1) and 75 in FHS (Supplementary Material, Table S3 and Fig. S2), allocated mostly to the *SLC2A9*-*WDR1* (4p16.1) and *ABCG2* regions (4q22) in both cohorts. These results are in line with the meta-analysis (6). The lead SNP associated with SUA was rs3733588 in both cohorts (Supplementary Material, Tables S2 and S3).

**Figure 1.**
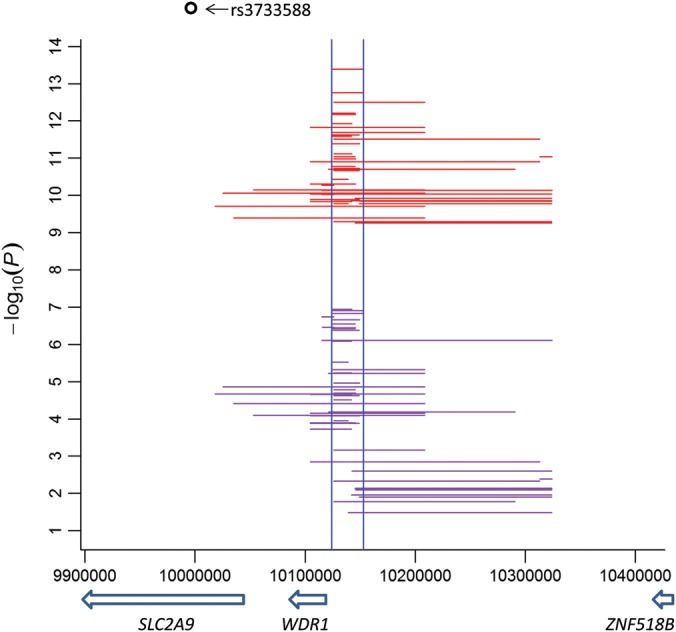
Genome-wide significant SNP pairs in ARIC (red) and their replication (*P*_*int*_ < 0.05) in FHS (purple). Each horizontal line represents an interaction between two SNPs located at the start and end of the line; two vertical lines mark the 30-kb window described in the main text; *y*-axis: interaction *P*-values in the −log_10_ scale; *x*-axis: genomic location in base pair (UCSC hg19/NCBI 37.3); arrow bar showing transcription direction and location of the gene (italic) below the bar; rs3733588 is the lead GWAS SNP.

Using the Bonferroni-corrected threshold of 3.8E−13 for a full pairwise genome scan in ARIC, we identified five significant epistatic SNP pairs that were well replicated in FHS when both SNPs were genotyped (as was the case for 3 of the 5 pairs, see Table 1). Each of the five pairs involved at least one marginal SNP (Supplementary Material, Table S2) and had no LD between the two SNPs. All interacting SNPs were located in an intergenic area between *WDR1* and *ZNF518B* within the 4p16.1 region, where the top four pairs of SNPs fell into a small window of <30 kb implicating a common epistatic signal upstream of rs3733588 (Fig. 1).

**Table 1.** Genome-wide significant (*P* < 3.8E−13) SNP pairs in ARIC and replication in FHS

chr	SNP_1_	pos_1_	SNP_2_	Pos_2_	Dist	LD (*r*^2^)	*P*_int_	*P*_int__FHS
4	**rs4697924**	10 124 239	rs731069	10 152 431	28.2	0.000	6.1E−14	NA
4	**rs4697924**	10 124 239	rs747357	10 152 878	28.6	0.000	2.2E−13	NA
4	**rs4697926**	10 124 567	rs731069	10 152 431	27.9	0.000	4.0E−14	1.5E−07
4	**rs4697926**	10 124 567	rs747357	10 152 878	28.3	0.000	1.7E−13	1.2E−07
4	**rs11722989**	10 126 139	**rs6845818**	10 208 794	82.7	0.002	3.1E−13	6.9E−04

chr—chromosome of a SNP pair; SNP_1_ (SNP_2_), pos_1_ (pos_2_)— name and position of the first (second) SNP; dist—distance in kb between two SNPs; LD (*r*^2^)— linkage disequilibrium between two SNPs; *P*_int_—*P*-value of the interaction test; *P*_int__FHS—interaction *P*-value of the SNP pair in FHS; NA—not directly replicated in FHS; SNPs in bold were genome-wide significant in GWAS in ARIC.

Using the genome-wide threshold of 5.9E−10 for interactions involving marginal SNPs (Materials and Methods), we further identified 83 significant pairs of SNPs all mapped to the 4p16.1 region, of which 45 pairs of interactions were directly replicated (i.e. both SNPs were genotyped with *P*_int_ <0.05) in FHS (Supplementary Material, Table S4). The 48 directly replicated SNP pairs (including the three in Table 1) were plotted in Figure 1, showing they were scattered mostly in the intergenic areas upstream of rs3733588.

**Figure 2.**
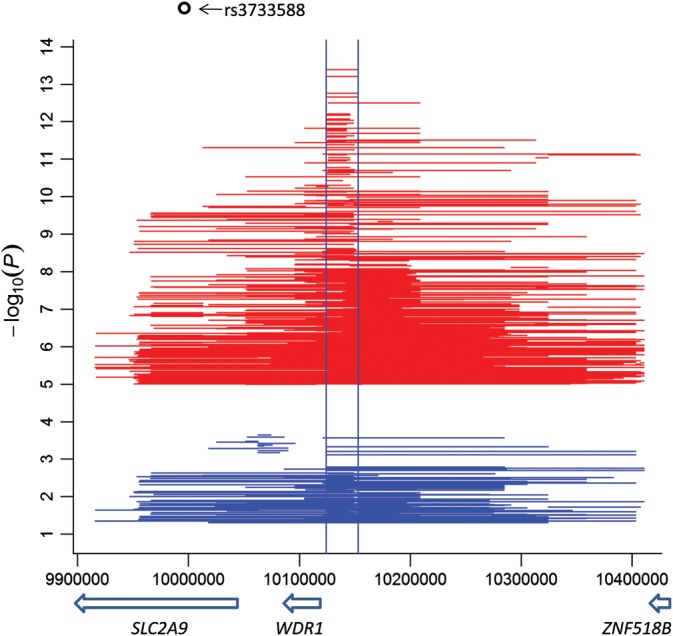
Local interactions in the 4p16.1 region (red) and those remaining significant (*P_int_* < 0.05) in conditional tests on the lead SNP rs3733588 (blue) in ARIC. Each horizontal line represents an interaction between two SNPs at the start and end locations; two vertical lines mark the 30-kb window described in the main text; *y*-axis: interaction *P*-values in the −log_10_ scale; *x*-axis: genomic location in base pair (UCSC hg19/NCBI 37.3); arrow bar showing transcription direction and location of the gene (italic) below the bar.

A further assessment of local interactions (i.e. two SNPs within 1 Mb with *P*_int_ < 1.0E−05) found the whole 4p16.1 region was enriched with interaction signals (917) in ARIC (Fig. 2) as well as FHS (Supplementary Material, Fig. S3). Outside of this region, we also observed a strong local interaction between rs2622621 and rs1564481 (both SNPs within *ABCG2* with *P*_int_ = 6.2E−11, distance = 30.3 kb, *r*^2^ = 0.23), which, however, was not replicated in FHS. Another SUA-associated gene tagged by local interactions in ARIC was *BCAS3* on chromosome 17: rs9914370 (*BCAS3*)–rs758596 (*TBX4*) (*P*_int_ = 6.0E−06, distance = 522.5 kb, *r*^2^ = 0.0) that was not replicated in FHS either.

We then performed conditional tests of the 917 local interaction pairs observed in the 4p16.1 region in ARIC by fitting the lead associated SNP rs3433588 (additive effect only) in the background and found 27% of them with *P*_int_ <0.05 across the region (Fig. 2). All the top five SNP pairs in Table 1 and 38 out of the 45 directly replicated significant SNP pairs (Fig. 1, Supplementary Material, Table S4) passed the conditional tests, suggesting they did not simply mirror the marginal effects of rs3433588. Similar conditional tests of the remaining marginal SNPs within the region also suggested multiple independent associations (*P* < 0.05) that were generally in low LD with the lead SNP rs3433588, except for several SNPs in relatively long range (e.g. >100 kb) LD (0.5 < *r*^2^ < 0.6) (Supplementary Material, Table S5 and Fig. S4).

Using the forward selection approach, we selected five independent marginal SNPs (i.e. rs3733588, rs874432, rs4697695, rs9291683 and rs11734783) capturing most marginal effects of the region, where the first three are within *SLC2A9* and the latter two are intergenic between *WDR1* and *ZNF518B*. Conditioning on the five selected marginal SNPs, we still found ∼10% (88 out of 917) of the 4p16.1 local interactions remained significant (*P*_int_ <0.05), including only one genome-wide significant pair (i.e. rs4697708–rs16895984, conditional *P*_int_ = 0.047, *r*^2^ = 0.122) (Supplementary Material, Table S6 and Fig. S5). A forward selection of the 10% significant local interactions led to three independent SNP pairs all located between *WDR1* and *ZNF518B* (Table 2). The selected five marginal SNPs and three SNP pairs jointly explained 6.0% of the SUA residual variance—1.5% more than that explained by the lead SNP alone but only 0.3% was contributed by the two marginal SNPs and three epistatic SNP pairs in the intergenic area with the remaining 1.2% owing to the two additional SNPs in *SLC2A9*. Nevertheless, without conditioning on the five marginal SNPs, the three epistatic SNP pairs could jointly explain 3% of the SUA residual variance.

**Table 2.** Statistical construction of the genetic structure of the 4p16.1 region

SNP_1_	pos_1_	SNP_2_	pos_2_	dist	LD (*r*^2^)	*P*-value	Variance explained (%)
rs3733588	9 997 303					6.9E−60	4.5
rs874432	9 920 606					1.4E−10	5.5
rs4697695	9 915 850					3.1E−04	5.7
rs9291683	10 324 160					4.6E−03	5.7
rs11734783	10 240 663					1.4E−02	5.9
rs731069	10 152 431	rs10939766	10 204 970	52.5	0.227	1.5E−02	5.8
rs4698000	10 277 467	rs11943276	10 403 545	126.1	0.042	1.7E−02	5.9
rs6813385	10 148 828	rs16894270	10 165 779	17.0	0.142	7.2E−03	6.0

only additive effects considered for first marginal SNPs; SNP_1_ (SNP_2_), pos_1_ (pos_2_)— name and position the first (second) SNP; dist—distance in kb between two SNPs; LD (*r*^2^)— linkage disequilibrium between two SNPs; *P*-value of a marginal SNP or interaction *P*-value of an epistatic SNP pair; Variance explained—SUA residual variance explained accumulated; blank cells: no information required.

We further imputed the 4p16.1 region (i.e. 9 900 000–10 400 000) in ARIC using the 1000 Genomes Project reference panel and tested whether the observed local interactions reflect a single untyped variant. Fourteen imputed SNPs had associations stronger than rs3733588, but the associations were not substantially different (Supplementary Material, Table S7). Using the forward selection approach described earlier, six independent imputed SNPs were selected: rs938558, rs4428284, rs4697695 (also typed), rs10489074, rs4481233 (also typed) and rs16895984 (also typed), where rs10489074 and rs16895984 are intergenic between *WDR1* and *ZNF518B* and the rest are within *SLC2A9*. Conditioning on the six selected imputed SNPs, 45 local interactions remained significant (*P*_int_ <0.05) (Supplementary Material, Table S8), most also appeared in Supplementary Material, Table S6 including the genome-wide significant rs4697708–rs16895984. These results suggested there were local interactions independent to marginal effects.

The genome-wide significant local interactions in the 4p16.1 region (Table 1 and Supplementary Material, Table S4, 42 unique epistatic SNPs) were analysed for enrichment of active regulatory regions using an online tool HaploReg (35). We found significant enrichments of enhancer regions in ENCODE (36) HepG2 (hepatocyte, 5.2-fold enrichment, *P* = 4.7E−5) and K562 (blood erythroblast, 5.2-fold enrichment, *P* = 5.0E−6) cell lines (Table 3). No similar significant enrichments were seen for ENCODE enhancer annotations for a variety of other ENCODE cell types (HUVEC umbilical vein endothelial cells, HMEC mammary epithelial cells, GM12878 B-lymphocytes, NHEK epidermal keratinocytes). The significant enrichments of enhancers in HepG2 and K562 cell lines were also observed in the analyses of GWAS marginal SNPs in the 4p16.1 region in both ARIC (Supplementary Material, Table S9) and FHS (Supplementary Material, Table S10).

**Table 3.** Enrichment of ENCODE enhancers by genome-wide significant local interactions in the 4p16.1 region in ARIC

Cell type		All enhancers				Strongest enhancers			
ID	Description	Obs.	Exp.	Fold	*P*-value	Obs.	Exp.	Fold	*P*-value
HepG2	Hepatocellular carcinoma	9	1.7	5.2	4.7E−05	5	0.6	8.3	3.4E−04
HUVEC	Umbilical vein endothelial cells	7	2.2	3.2	6.0E−03	2	1.1	1.8	3.1E−01
K562	Leukaemia	11	2.1	5.2	5.0E−06	10	0.7	15.2	<1.0E−06
HMEC	Mammary epithelial cells	7	3	2.3	3.0E−02	2	1.2	1.7	3.4E−01
GM12878	B-lymphocyte lymphoblastoid	5	2.2	2.3	6.6E−02	5	0.8	6.2	1.2E−03
NHEK	Epidermal keratinocytes	7	2.7	2.6	1.7E−02	4	1.2	3.3	3.1E−02

Obs.: observed; Exp.: expected; Fold: fold of enrichment calculated as Obs./Exp.; 42 unique epistatic SNPs from Table 1 and Supplementary Material, Table S4 used in the enrichment analysis.

Closer examination of the chromatin states predicted using the software chromHMM (37) also suggested distinct enhancer activity signals in the 4p16.1 region (the chromHMM category plot, Supplementary Material, Fig. S6), including several strong enhancers located in the two intergenic areas flanking *WDR1* in the HepG2 cell line. Consistent with this, transcription factor binding-site clusters and various other features of functionally active HepG2 chromatin were also found in these intergenic areas (Supplementary Material, Fig. S6). The intergenic area between *WDR1* and *ZNF518B* appears to be bound by transcription factors and RNA polymerase II in cell lines including HepG2 and K562 (Supplementary Material, Fig. S7). Similarly, the intergenic area between *SLC2A9* and *WDR1* is actively transcribed in multiple cell lines including HepG2 and K562, again consistent with active enhancers in this region (Supplementary Material, Fig. S8). Near the 30-kb window marked by the top five SNP interactions (Table 1 and Fig. 1), we found strong chromatin interactions corresponding to the enhancers in the area in the ChIA-PET (Chromatin Interaction Analysis by Paired-End Tag Sequencing) data from the K562 cell line, including a chromatin interaction involving the transcription start site (TSS) of *WDR1* and mediated by RNA polymerase II (Supplementary Material, Fig. S7). To investigate whether ChIA-PET interactions are commonly enriched in other GWAS regions, we sorted 17 680 marginal SNPs currently available from the GWAS Catalog (38) into 8817 regions each encompassing 25 kb and then counted ChIA-PET interactions within a distance of 100 kb flanking the middle point of each sorted region. We found <4% (352 out of 8817) of the sorted GWAS regions had more ChIA-PET interactions than the *SLC2A9* region (i.e. chr4: 10080000–10105000, with 96 ChIA-PET interactions). This empirical analysis places the observed *SLC2A9* enrichment for interactions within a small proportion of known complex trait loci identified by GWAS.

We further examined local interactions in the 4p16.1 region in ARIC female and male samples separately following the same analysis procedure (Supplementary Material, Fig. S9). Local interactions in the 30-kb window appeared relatively consistently in both genders suggesting a common enhancer activity. The most striking difference was that females had very strong (−log_10_*P*_int_ > 14) long range interactions between two *WDR1* SNPs (rs4604059 and rs12498927) and two intergenic SNPs (rs7681212 and rs11943276) near *ZNF518B*, which did not appear in males (Supplementary Material, Fig. S9).

## DISCUSSION

Using ARIC and FHS, we were able to detect genome-wide significant epistasis in SUA (Table 1) based on a stringent Bonferroni-corrected threshold (20). Additional searches focusing on marginal SNP interactions with a relaxed significance threshold found more significant epistatic signals, all within the 4p16.1 region covering *SLC2A9* (Fig. 1 and Supplementary Material, Table S4). The significant epistatic signals identified in ARIC were well replicated in FHS, either exactly as the same SNP pair if both SNPs were genotyped (Fig. 1), or at the regional level (Fig. 2 and Supplementary Material, Fig. S3) (23–25,39).

The observations were reinforced by abundant and widespread local interactions within the 4p16.1 region in both ARIC and FHS (with ∼103 000 SNPs less than ARIC) (Fig. 2 and Supplementary Material, Fig. S3). The conditional analysis results based on the lead SNP rs3733588 showed that a number of local interactions and marginal SNPs were statistically independent, supporting the hypothesis of multiple variants residing in the region (Fig. 2 and Supplementary Material, Fig. S4). This differs from the report of only one associated SNP within *SLC2A9* from the meta-analysis (6), i.e. rs12498742 that is 53 kb away from rs3733588, possibly because (a) rs12498742 did not pass the quality control in this study and (b) the meta-analysis placed an additional requirement of SNP effect size reduction (≤20%) to claim an independent signal in the conditional tests.

To fully assess the impact of marginal effects on local interactions in the region, we forward-selected five marginal SNPs as the additive genetic background and still found a substantial number of local interactions significant in the new conditional tests, most involving at least one epistatic SNP located in the intergenic areas flanking the *WDR1* gene (Supplementary Material, Fig. S5), suggesting there might reside some regulatory elements. We showed that these local interactions did not reflect the effects of a single imputed SNP. The ‘final’ forward selection model of the five marginal SNPs and three SNP pairs further emphasized the intergenic area between *WDR1* and *ZNF518B*, covering the two marginal SNPs and all the three epistatic SNP pairs.

Functional analyses provide strong evidence that the epistatically interacting SNPs are unusually enriched at enhancers active mainly in hepatic and precursor red blood cell types implicated in SUA (Table 3). Despite the fact that many regions of the genome may show enhancer activity in some cell type at some time, identifying cell-type-specific enhancers by integrating GWAS and epigenetic signals has become increasingly useful for functionally studying complex traits (40). The HaploReg enrichment test applied is a statistically rigorous approach for this purpose that uses a rigorously defined genomic background given all the SNPs genotyped (35). To our knowledge, the present study is the first in applying this approach to elucidating the biological basis of epistatically interacting loci and generated testable hypotheses for follow-up functional work by experimental biologists. Particularly in the *WDR1*-*ZNF518B* intergenic area, statistical interactions, enhancers, chromatin interactions between TSS of *WDR1* and the enhancers collectively suggest complex mechanisms regulating *SLC2A9* function, which may potentially contribute to the *SLC2A9*-mediated effect on gender difference in SUA levels (6). The observations that both intergenic areas flanking *WDR1* are actively transcribed with many transcripts overlapping or adjacent to each other lead to the hypothesis that *SLC2A9* and *WDR1* may be co-transcribed or share transcription regulatory machinery. The hypothesis is intriguing as *SLC2A9* gene expression may be regulated by enhancers directly targeting *SLC2A9* and/or indirectly regulated by other enhancer(s) via *WDR1* transcription. Further work is needed to test these hypotheses and dissect the regulatory mechanisms.

In this study, we detected no genome-wide significant epistatic signals other than those in the 4p16.1 region, reinforcing the impression that single-GWAS populations are generally underpowered for studying epistasis (24). Indeed, even in conventional GWAS, *GCKR* was the only locus other than *SLC2A9* and *ABCG2* detected significantly in ARIC (suggestively in FHS). Hence, at the level of single-GWAS populations, searching for marginal SNP interactions and local interactions under relaxed significance thresholds are perhaps more realistic approaches. For example, local interactions also captured *ABCG2* and *BCAS3* despite not being directly replicated in FHS. In fact, a suggestive local interaction pair of rs2725227 and rs2725222 (distance = 14 kb, *P*_int_ was 1.2E−05 in ARIC and 9.6E−03 in FHS) was near *ABCG2*, with both SNPs were located in *PKD2*, a candidate causal locus of polycystic kidney disease (41) and regulator of SUA levels (42).

Local interactions were not found for all GWAS loci, e.g. no local interactions were observed in the *GCKR* locus at all in this and our previous study of eight metabolic traits (24). Abundant local interactions seem more likely to be seen in regions with greater genetic heterogeneity, e.g. the human leukocyte antigen (HLA) region for auto-immune diseases (43,44), the 11q23.3 region for lipid traits (24). We also observed local interactions in other regions not harbouring associated variants across the genome (results not shown) but concentrated on the 4p16.1 region in this study. Considering the difficulty in differentiating haplotype effects from true interactions statistically (21,24), future functional work is needed to decipher any haplotypes like those in HLA (45) or real functional interactions (34,46,47).

In this and our previous studies of epistasis in quantitative traits (21,24,25,39), the trait values used for testing epistasis were the resultant residuals of a mixed model following an adapted GRAMMAR approach (48) to correct for polygenic effects and covariates including the first 10 principal components computed from the genomic relationship matrix to account for relatedness in samples. It is known that the GRAMMAR approach is conservative in conventional GWAS that consider only additive effects and the conservativeness increases as population substructure and trait heritability increase (48,49). While it is unclear whether the GRAMMAR approach remains conservative in epistatic models, the adapted GRAMMAR approach (i.e. accounting for relatedness simultaneously) seems to be not conservative in our previous study of epistasis in SUA in isolated populations (25). Nonetheless, as a precaution, one could allow additional epistatic signals out of BiForce screening to enter the full model tests and apply predefined thresholds afterwards. Such full model tests are essential to assess the screening results as BiForce uses approximate statistical tests for interactions and treats each pair of SNPs independently (21).

In summary, pairwise genome-wide screening for epistasis in SUA allowed us to detect abundant local interactions in the 4p16.1 region that highlighted the functional complexity of the region and provided compelling insights into potential mechanisms regulating *SLC2A9* functions.

## MATERIALS AND METHODS

This study was approved by the institutional review board of the West of Scotland Research Ethics Service of NHS in the UK. The GWAS data of the ARIC and FHS study cohorts are provided by the NIH Database of Genotype and Phenotype via specific Data Use Certifications issued by the Data Access Committee of the National Heart, Lung and Blood Institute. Both study cohorts have been described in detail elsewhere (50–53). Only individuals with European ancestry of the two study cohorts were used in this study. Both ARIC and FHS were approved by corresponding local ethics committees and obtained written informed consent from the study participants. ARIC was genotyped with the Affymetrix 6.0 SNP chip and the FHS cohort with Affymetrix 500K and Affymetrix 50K SNP chips.

A common protocol was used to perform quality control of the genotype data in both cohorts using the GenABEL package (54) implemented in R (http://www.r-project.org/): individual call rate at 97%, SNP call rate at 95%, minor allele frequency at 2%, *P*-value for deviation from Hardy–Weinberg equilibrium at 1.0E−10, false discovery rate for unacceptably high individual heterozygosity at 0.01. SUA in ARIC was corrected for sex, age, body mass index (BMI), serum creatinine, hypertension treatment and sample centre. SUA in FHS was corrected for sex, age, BMI, creatinine, hypertension treatment, renal disease status and generation (SUA in generations 2 and 3 samples measured at their second and first visit, respectively). To control relatedness, individuals that were outliers of the first three principal components computed from the identity-by-state matrix constructed using GenABEL were removed. In addition, subjects younger than 18 years old, or with BMI >50, or with creatinine beyond the range of 3 SD of the population mean were removed from the study. After quality control, 9172 (4884 females) and 5538 (2951 females) samples, 514 662 and 410 947 autosomal SNPs were analysed in ARIC and FHS, respectively (Supplementary Material, Table S1).

Genome scans were performed for each cohort as follows: (a) the identity-by-state matrix was reconstructed and the first ten principal components were calculated and stored; (b) SUA was adjusted for covariates correspondingly and normalized using the GenABEL *rntransform* function and then adjusted for polygenic effects and the first ten principal components to account for relatedness using the mixed model-based *polygenic* function where the polygenic heritability was computed (Supplementary Material, Table S1) and the resultant environmental residuals (i.e. *pgresidualY*) were used as the actual trait values for association tests (48); (c) conventional GWAS analyses (i.e. assuming additive effects only) were performed using the GenABEL *mmscore* function (49) and the consensus threshold (*P* = 5.0E−08) (55) was used to identify marginal SNPs; (d) full pairwise genome scans using BiForce that utilizes bitwise data structures and advanced algorithms to allow high-throughput detection of epistasis (12). Genome-wide significant thresholds were derived based on the Bonferroni adjustment of actual number of tests as previously described (12,21), i.e. with 514 662 SNPs and 166 marginal SNPs identified (Supplementary Material, Table S2) in ARIC, the thresholds were 3.8E−13 (*P* =0.05/(514662 × (514662–1)/2)) for SNP pairs identified from the full pairwise genome scan and 5.9E−10 (*P* = 0.05/((514662–1) × 166)) for SNP pairs involving at least one marginal SNP. We adopted the threshold of 1.0E−05 for local interactions derived previously based on permutation (24).

Significant epistatic SNP pairs were tested for replication in FHS at the SNP level only for simplicity, i.e. claiming a replication of an epistatic pair only if both SNPs were genotyped and with *P*_int_ < 0.05 in FHS (24). Conditional tests were carried out by fitting one or multiple marginal SNPs as fixed effects in the background and then each of other SNPs or SNP pairs individually in the same way(s) as used in the genome scans and considering the SNP or SNP pair statistically independent if the conditional *P*/*P*_int_ < 0.05. The forward selection approach was used when multiple independent associations were available in the conditional tests: to select the most associated SNP or SNP pair (i.e. with the lowest conditional *P*/*P*_int_), fit into the background and test the remaining, repeating until no more significant conditional associations were found. Variance explained was calculated using the *polygenic* function with marginal SNPs or SNP pairs fitted as fixed effects.

We imputed the 4p16.1 region (from 9900 to 10400 kb) based on 9172 samples and 260 typed SNPs in ARIC using IMPUTE2 (56) and the 1000 Genomes Project reference panel (phase1 integrated variant set v3). We used SNPTEST (v2.5) (57) to test associations of 2610 imputed SNPs (minor allele frequency >0.01) with the same SUA trait in the frequentist additive model using genotype dosages. We used PLINK2 (https://www.cog-genomics.org/plink2/) to take the best genotypes of the imputed SNPs and then performed forward selection and conditional tests in R as described earlier.

GWAS marginal SNPs and genome-wide significant epistatic SNPs within the 4p16.1 region were analysed for enrichment of ENCODE (36) cell-type-specific enhancers using the online tool HaploReg (http://compbio.mit.edu/HaploReg) that tests enrichment based on a rigorously defined genomic background (i.e. all the SNPs genotyped) (35), with LD information (*r*^2^>0.8) from the 1000 Genomes Project and a background set of Affymetrix 6.0 SNPs. ANNOVAR (58) and UCSC genome browser (59) were used for functional annotation of SNPs within the region to identify regulatory signals associated with these loci. Enlight (http://enlight.usc.edu) was used to visually inspect the relationship between LD and regulatory signals.

## SUPPLEMENTARY MATERIAL

Supplementary Material is available at *HMG* online.

## FUNDING

W.H.W., C.A.S. and C.S.H. are supported by the UK Medical Research Council University Unit Strategic Partnership Funding to MRC Human Genetics Unit, University of Edinburgh. W.H.W. is partially funded by Higher Education Funding Council for England (HEFCE). Y.G. and K.W. are supported by NIH Grant R01 HG006465. BBSRC Travel Grant (BB/K004964/1) initiated the collaboration with K.W. Funding for open access charge: the UK Medical Research Council Core Fund. Funding to pay the Open Access publication charges for this article was provided by the UK Medical Research Council University Unit Strategic Partnership Funding to MRC Human Genetics Unit, University of Edinburgh.

## Supplementary Material

Supplementary Data
