# Supplementary material for: Abundant local interactions in the 4p16.1 region suggest functional mechanisms underlying SLC2A9 associations with human serum uric acid
Source: Hum Mol Genet. 2014 May 12;23(19):5061–8. doi: 10.1093/hmg/ddu227 (PMC4159153; doi:10.1093/hmg/ddu227)
Supplement: Supplementary Data [file supp_23_19_5061__index.html]

Abundant local interactions in the 4p16.1 region suggest functional mechanisms underlying SLC2A9 associations with human serum uric acid — Abundant local interactions in the 4p16.1 region suggest functional mechanisms underlying SLC2A9 associations with human serum uric acid — Supplementary Data 

# Abundant local interactions in the 4p16.1 region suggest functional mechanisms underlying *SLC2A9* associations with human serum uric acid

## Supplementary Data

Supplementary Data

**Files in this Data Supplement:**

- Supplementary Data - Pdf file
- Supplementary Tables - xlsx file
