# Supplementary material for: Abundant local interactions in the 4p16.1 region suggest functional mechanisms underlying SLC2A9 associations with human serum uric acid
Source: Hum Mol Genet. 2014 May 12;23(19):5061–8. doi: 10.1093/hmg/ddu227 (PMC4159153; doi:10.1093/hmg/ddu227)
Supplement: Supplementary Data [file supp_ddu227_ddu227supp.pdf]

Supporting Information

Table S1: Summary information of the ARIC and FHS cohorts

Table S2: Genome-wide significant SNPs detected in GWAS of ARIC

Table S3: Genome-wide significant SNPs detected in GWAS of FHS

Table S4: Genome-wide significant interactions involving marginal SNPs in ARIC and their direct replication in FHS

Table S5: Conditional test P values of the genome-wide significant SNPs in the 4p16.1 region in ARIC and their LD values with the lead SNP

Table S6: Local interactions in the 4p16.1 region significant in tests conditional on the five selected marginal SNPs in ARIC

Table S7: Significantly associated imputed SNPs ($P < 5.0e-08$) in SNPTEST analysis of the 4p16.1 region in ARIC

Table S8: Local interactions in the 4p16.1 region significant in tests conditional on the six selected marginal imputed SNPs in ARIC

Table S9: Enrichment of ENCODE enhancers by 155 GWAS marginal SNPs in the 4p16.1 region in ARIC

Table S10: Enrichment of ENCODE enhancers by 72 GWAS marginal SNPs in the 4p16.1 region in FHS

Figure S1: Manhattan and Q-Q plots of the conventional GWAS of SUA in ARIC. Red horizontal line represents the consensus genome-wide significance threshold.

Figure S2: Manhattan and Q-Q plots of the conventional GWAS of SUA in FHS. Red horizontal line represents the consensus genome-wide significance threshold.

Figure S3: Local interactions in the 4p16.1 region in FHS. Each horizontal line represents an interaction between two SNPs located at the start and end of the line; two vertical lines marks the 30 kb window described in the main text; y axis: interaction P values in the -$\log_{10}$ scale; x axis: genomic location in base pair; arrow bar showing transcription direction and location of the gene (italic) below the bar.

Figure S4: All marginal SNPs in the 4p16.1 region in ARIC, their LD with the lead SNP rs3733588 colored in purple and the recombination rate distribution in the region. left y axis: SNP association P values in the -$\log_{10}$ scale; right y axis: recombination rate; x axis: genes (italic, arrow bar showing transcription direction) and genomic location in Mb.

Figure S5: Local interactions in the 4p16.1 region remained significant ($P_{int} < 0.05$) in the tests conditional on five selected marginal SNPs in ARIC. Each horizontal line represents an interaction between two SNPs located at the start and end of the line; two vertical lines marks the 30 kb window described in the main text; y axis: conditional P values in the -$\log_{10}$ scale; x axis: genomic location in base pair; arrow bar showing transcription direction and location of the gene (italic) below the bar.

Figure S6: Chromatin states of the 4p16.1 region showing categorical classification of regulatory elements and UCSC genome browser data tracks of ENCODE data in the region (all scores are normalized from 0 to 1000). The wgEncodeBroadHmmHepg2HMM data track shows chromatin states in the region of HepG2 cells characterized using the ChromHMM software, including two strong enhancer zones flanking the *WDR1* gene; the wgEncodeRegTfbsClusteredV3 data track shows transcription factor binding site cluster assayed by ChIP-seq; the wgEncodeOpenChromChipHepg2CtcfPk data track shows DNase-seq signal peaks in

HepG2 cells marking open chromatin flanking *WDR1*; the wgEncodeBroadHistoneHepg2H3k27acStdPk data track displays active regions in HepG2 cells by histone mark H3K27Ac.

Figure S7: RNA sequencing signal and alignment, chromatin states and ChIA-PET alignment in the intergenic area between *WDR1* and *ZNF518B*. Chromatin states characterized by chromHMM suggest multiple enhancers in the area; RNA sequence alignment shows the intergenic area is actively transcribed in multiple cell lines including HepG2 and K562; The ChIA-PET alignment of data from K562 cells mediated by RNA polmerase II shows strong chromatin interactions (dark color) corresponding to enhancers displayed in chromHMM.

Figure S8: RNA sequencing signal and alignment and chromatin state classification in the intergenic area between *SLC2A9* and *WDR1*. RNA sequence alignment suggests the intergenic area is actively transcribed in multiple cell lines including HepG2. The chromatin state classification suggests multiple enhancers in the area.

Figure S9: Gender specific local interactions in the 4p16.1 region (red: female, 4884 samples; blue: male, 4288 samples) in ARIC. Each horizontal line represents an interaction between two SNPs at the start and end locations; two vertical lines marks the 30 kb window described in the main text; y axis: interaction P values in the $-\log_{10}$ scale; x axis: genomic location in base pair (UCSC hg19/NCBI 37.3); arrow bar showing transcription direction and location of the gene (italic) below the bar; rs3733588 is the lead GWAS SNP in female samples (P = 5.2E-65) and the sixth strongest associated SNP in male samples (P = 2.1E-17; P = 5.8E-18 in the lead SNP rs7442295).

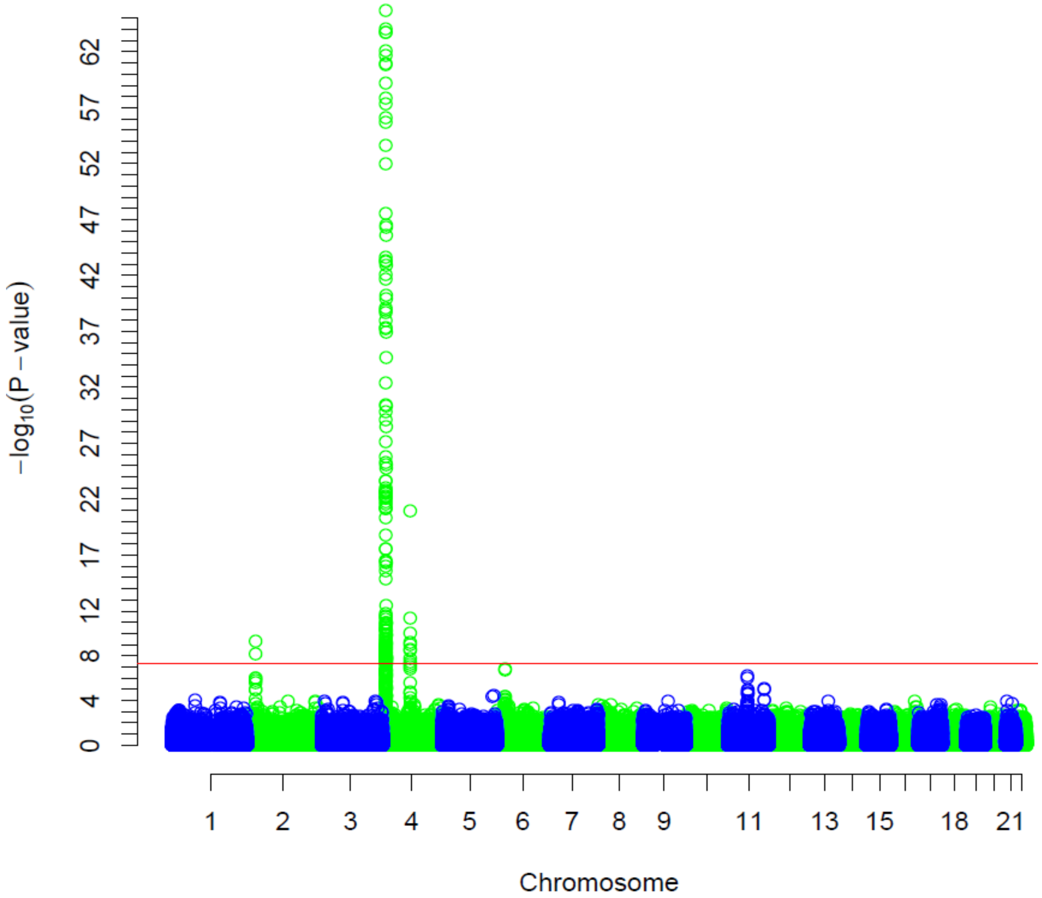

−log10(P−value)
0
4
8
12
17
22
27
32
37
42
47
52
57
62
1
2
3
4
5
6
7
8
9
11
13
15
18
21
Chromosome

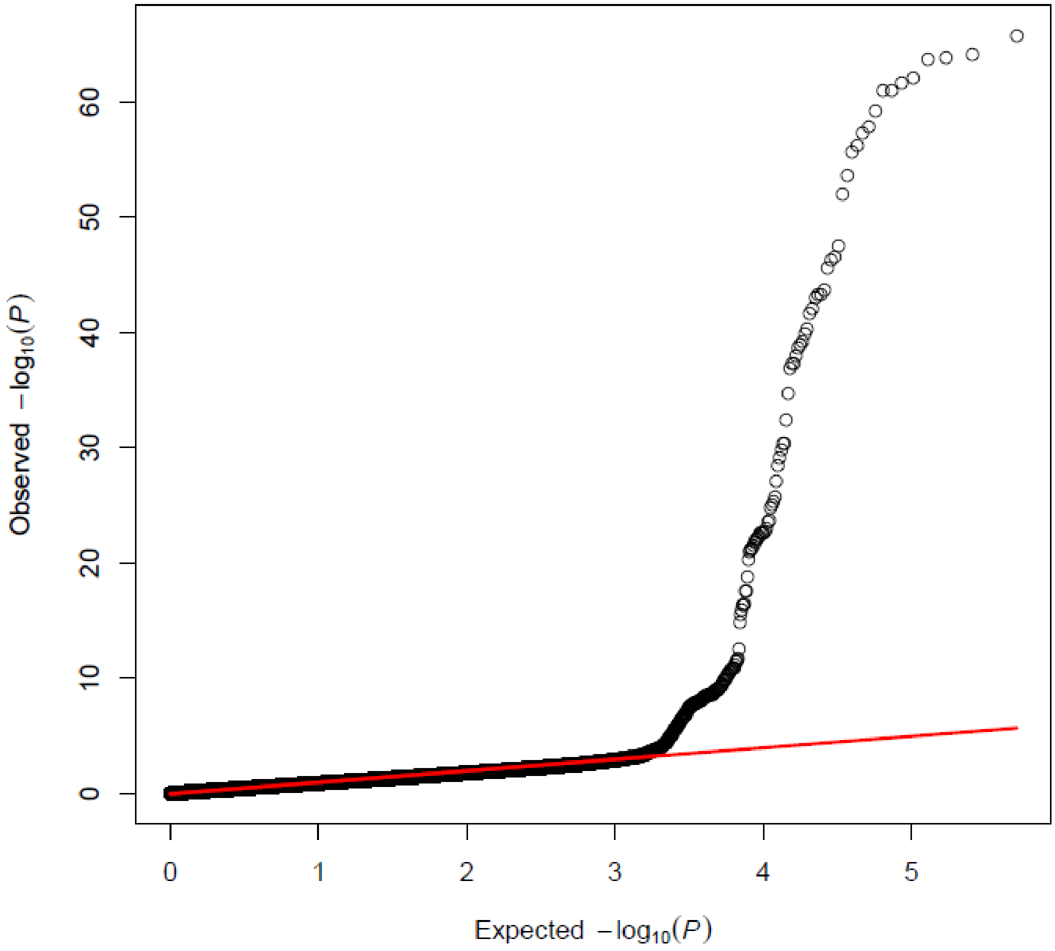

Observed −log10(P)
0
10
20
30
40
50
60
0
1
2
3
4
5
Expected −log10(P)

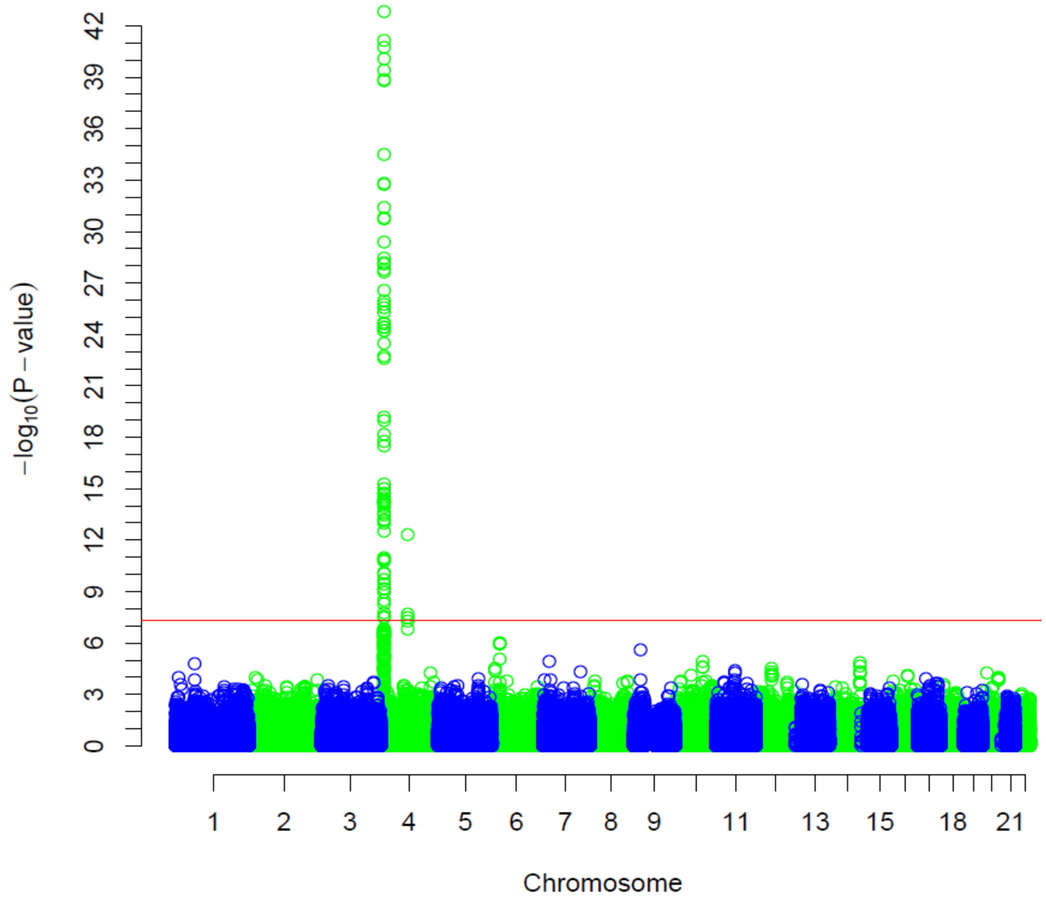

−log10(P−value)
0
3
6
9
12
15
18
21
24
27
30
33
36
39
42
1
2
3
4
5
6
7
8
9
11
13
15
18
21
Chromosome

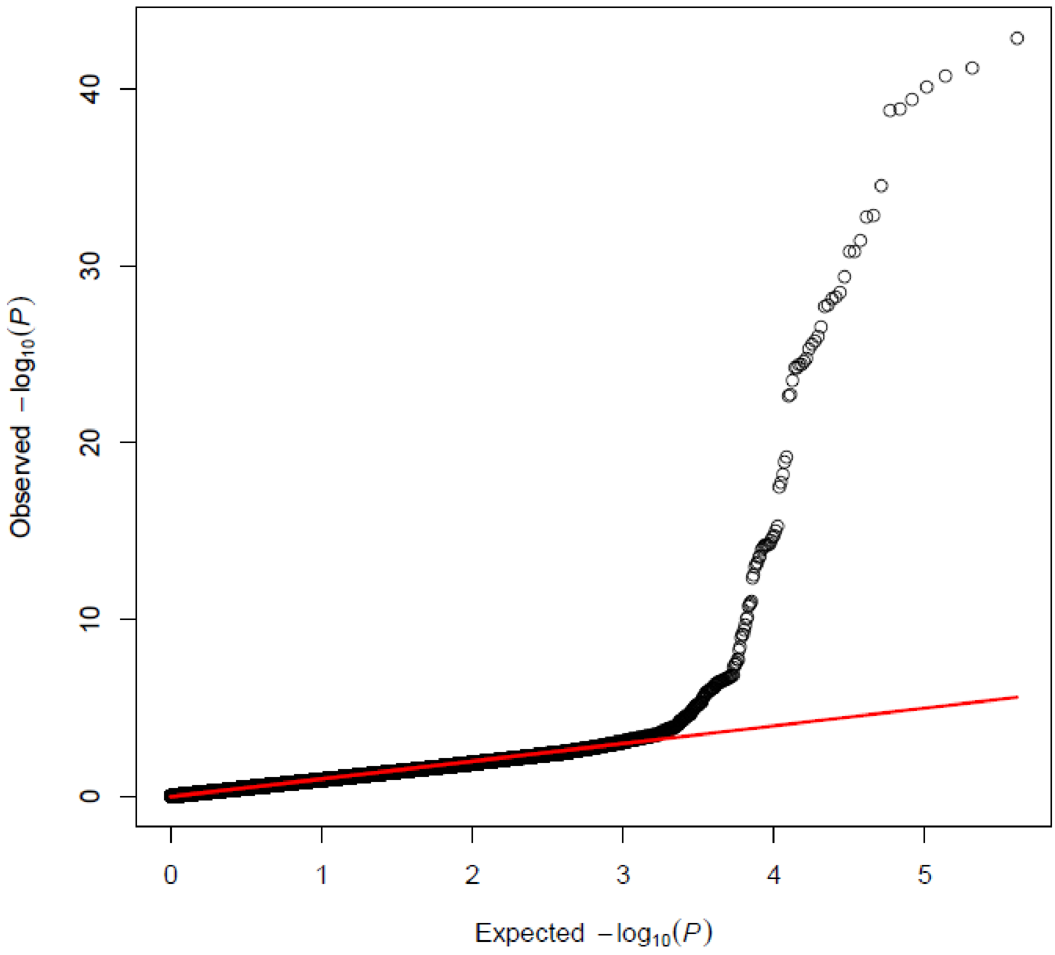

Observed −log10(P)
0
10
20
30
40
0
1
2
3
4
5
Expected −log10(P)

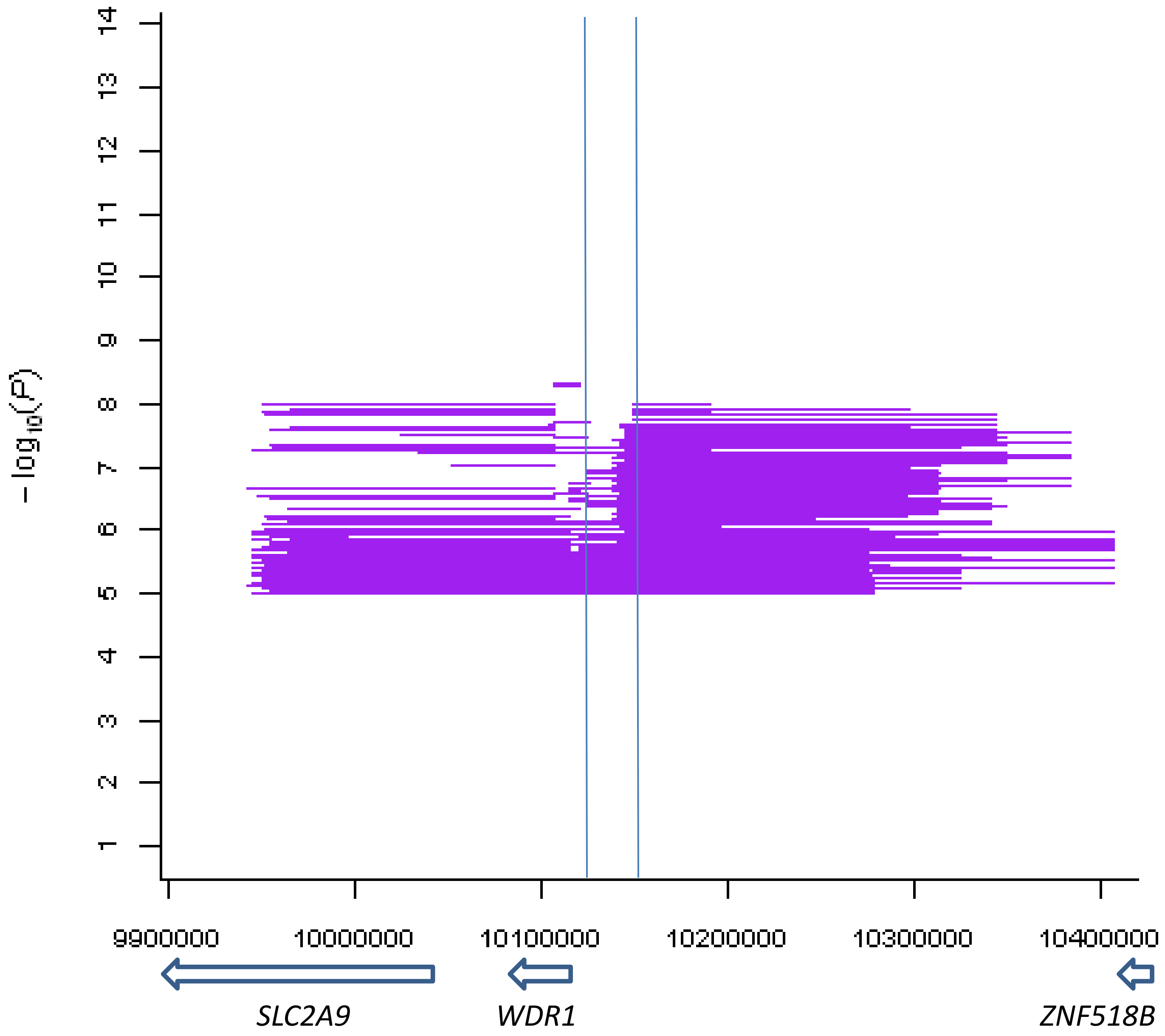

14
13
12
11
10
9
8
7
6
5
4
3
2
1
− log10(P)
9900000
10000000
10100000
10200000
10300000
10400000
SLC2A9
WDR1
ZNF518B

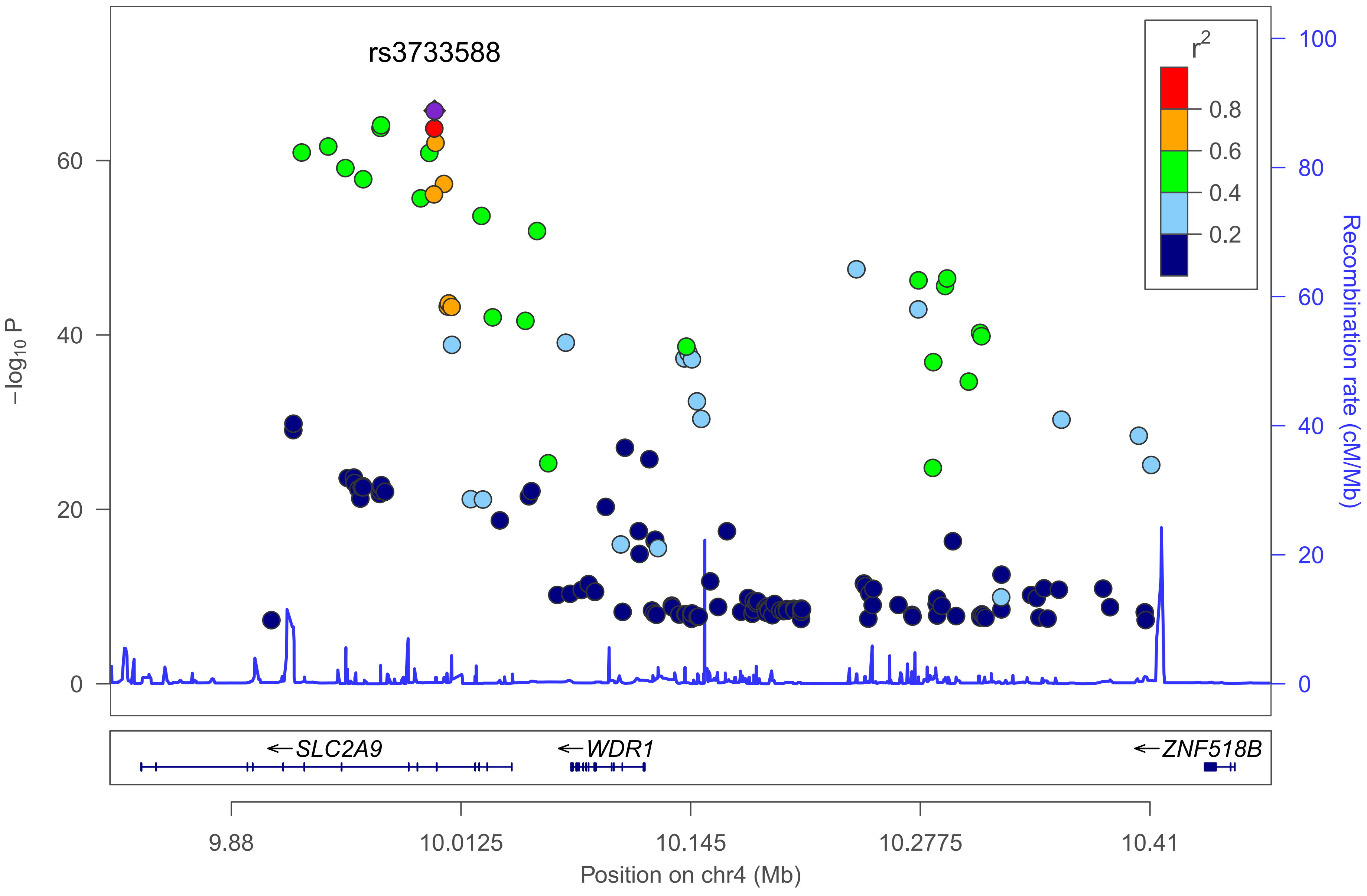
rs3733588
r2
0.8
0.6
0.4
0.2
−log10 P
60
40
20
0
100
80
60
40
20
0
Recombination rate (cM/Mb)
←SLC2A9
←WDR1
←ZNF518B
9.88
10.0125
10.145
10.2775
10.41
Position on chr4 (Mb)

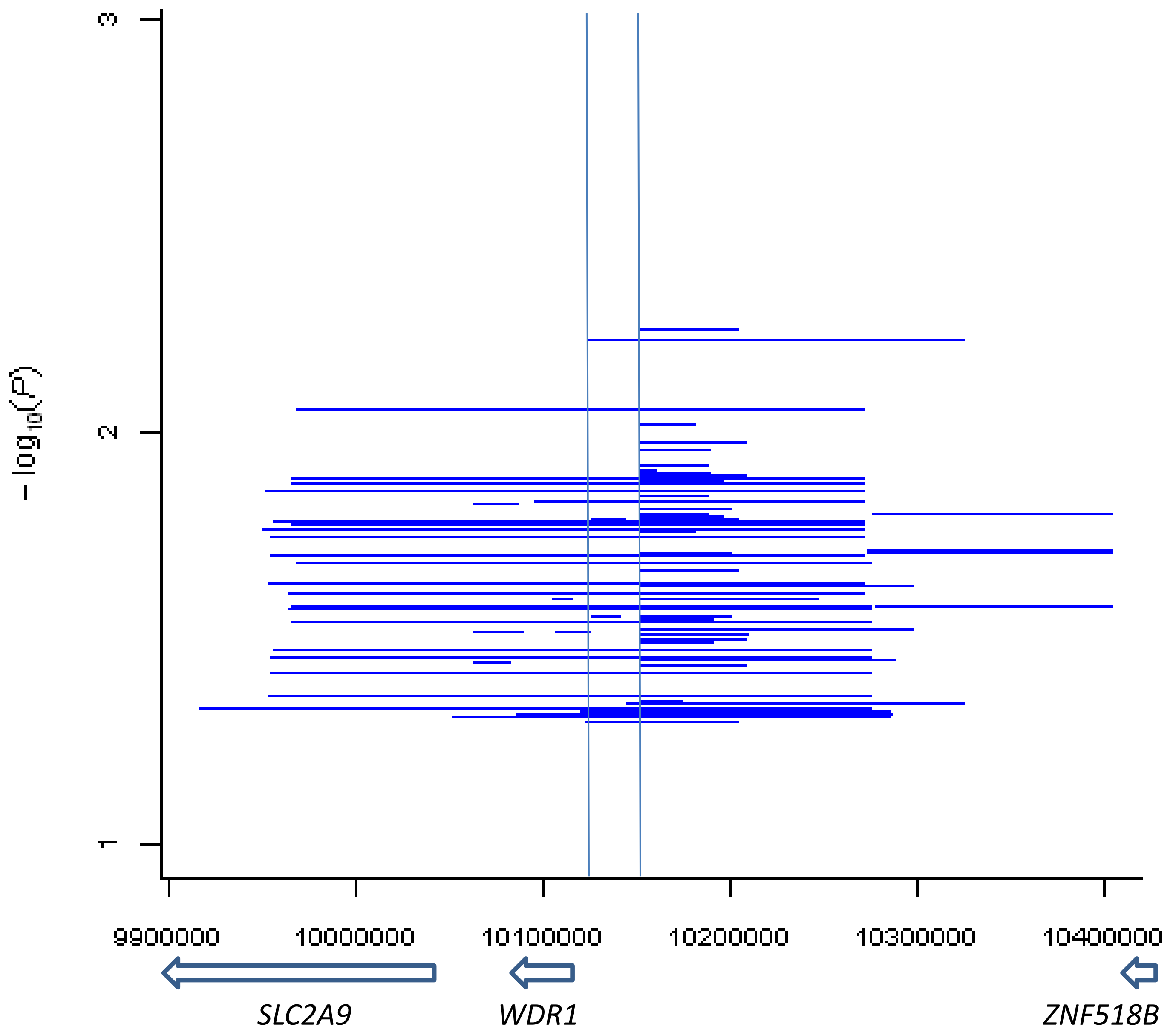

−log10(P)
3
2
1
9900000
10000000
10100000
10200000
10300000
10400000
SLC2A9
WDR1
ZNF518B

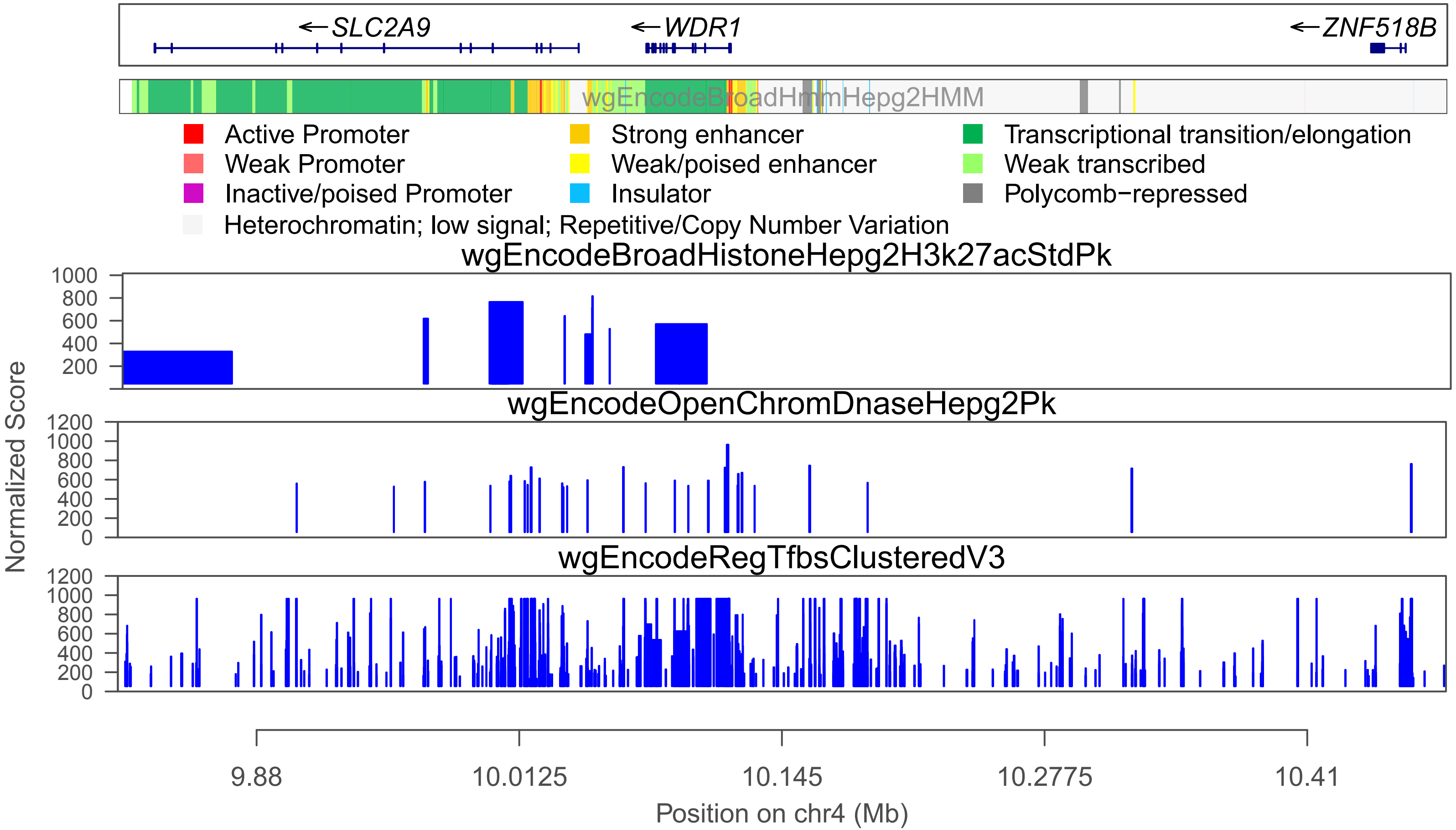

←SLC2A9
←WDR1
←ZNF518B
wgEncodeBroadHmmHepg2HMM
Active Promoter
Weak Promoter
Inactive/poised Promoter
Strong enhancer
Weak/poised enhancer
Insulator
Transcriptional transition/elongation
Weak transcribed
Polycomb−repressed
Heterochromatin; low signal; Repetitive/Copy Number Variation
wgEncodeBroadHistoneHepg2H3k27acStdPk
1000
800
600
400
200
wgEncodeOpenChromDnaseHepg2Pk
1200
1000
800
600
400
200
0
wgEncodeRegTfbsClusteredV3
1200
1000
800
600
400
200
0
Normalized Score
9.88
10.0125
10.145
10.2775
10.41
Position on chr4 (Mb)

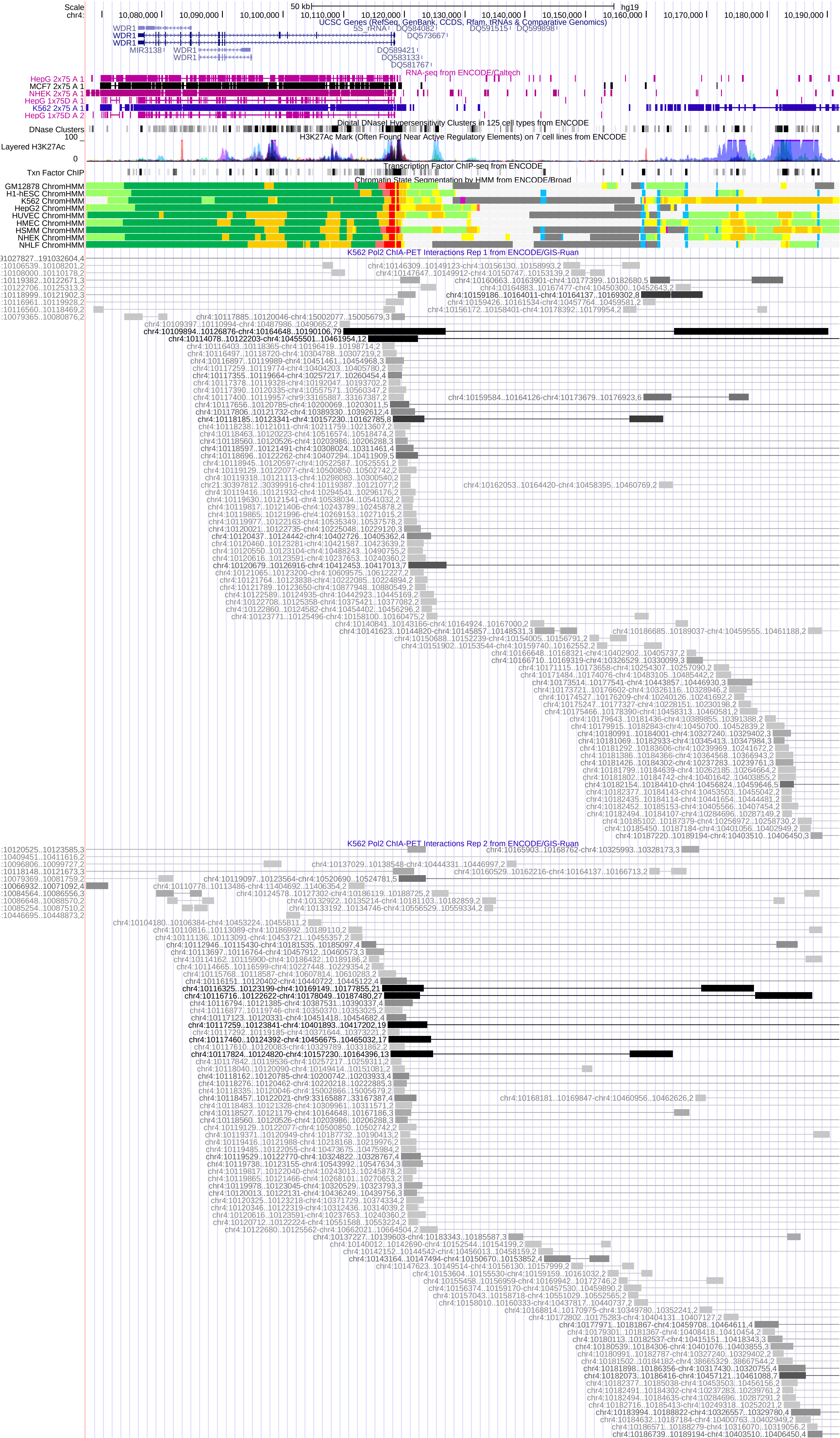

Scale
chr4:
50 kb
hg19
10,080,000
10,090,000
10,100,000
10,110,000
10,120,000
10,130,000
10,140,000
10,150,000
10,160,000
10,170,000
10,180,000
10,190,000
UCSC Genes (RefSeq, GenBank, CCDS, Rfam, tRNAs & Comparative Genomics)
WDR1
MIR3138
5S_rRNA
DQ584082
DQ573667
DQ591515
DQ599898
DQ589421
DQ583133
DQ581767
RNA-seq from ENCODE/Caltech
HepG 2x75 A 1
MCF7 2x75 A 1
NHEK 2x75 A 1
HepG 1x75D A 1
K562 2x75 A 1
HepG 1x75D A 2
DNase Clusters
Digital DNaseI Hypersensitivity Clusters in 125 cell types from ENCODE
100
H3K27Ac Mark (Often Found Near Active Regulatory Elements) on 7 cell lines from ENCODE
Layered H3K27Ac
0
Txn Factor ChIP
Transcription Factor ChIP-seq from ENCODE
Chromatin State Segmentation by HMM from ENCODE/Broad
GM12878 ChromHMM
H1-hESC ChromHMM
K562 ChromHMM
HepG2 ChromHMM
HUVEC ChromHMM
HMEC ChromHMM
HSMM ChromHMM
NHEK ChromHMM
NHLF ChromHMM
K562 Pol2 ChIA-PET Interactions Rep 1 from ENCODE/GIS-Ruan
chr4:10109894..10126876-chr4:10164648..10190106,79
chr4:10114078..10122203-chr4:10455501..10461954,12
chr4:10118185..10123341-chr4:10157230..10162785,8
chr4:10159186..10164011-chr4:10164137..10169302,8
chr4:10120679..10126916-chr4:10412453..10417013,7
K562 Pol2 ChIA-PET Interactions Rep 2 from ENCODE/GIS-Ruan
chr4:10116325..10123199-chr4:10169149..10177855,21
chr4:10116716..10122622-chr4:10178049..10187480,27
chr4:10117259..10123841-chr4:10401893..10417202,19
chr4:10117460..10124392-chr4:10456675..10465032,17
chr4:10117824..10124820-chr4:10157230..10164396,13

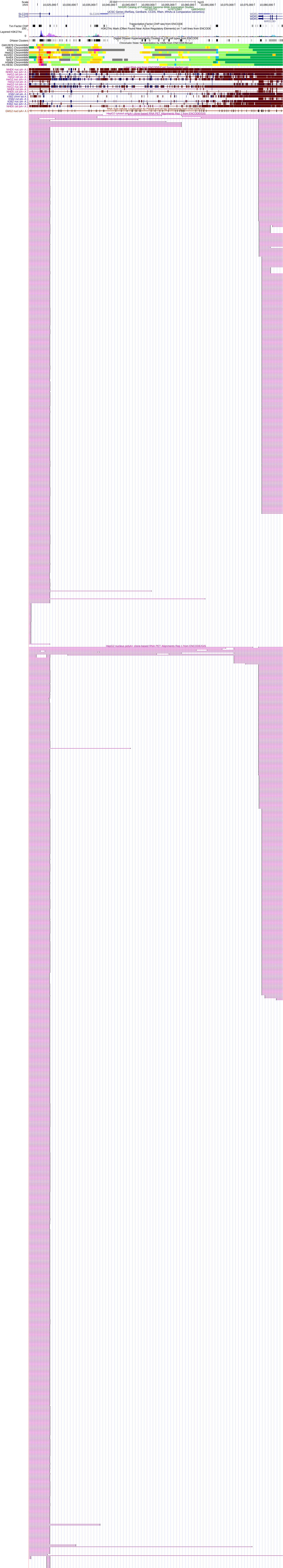
Scale
chr4:
20 kb
hg19
10,025,000
10,030,000
10,035,000
10,040,000
10,045,000
10,050,000
10,055,000
10,060,000
10,065,000
10,070,000
10,075,000
10,080,000
NHGRI Catalog of Published Genome-Wide Association Studies
rs733175
rs7671266
rs6834555
UCSC Genes (RefSeq, GenBank, CCDS, Rfam, tRNAs & Comparative Genomics)
SLC2A9
SLC2A9
SLC2A9
WDR1
WDR1
WDR1
MIR3138
Transcription Factor ChIP-seq from ENCODE
Txn Factor ChIP
100 _
H3K27Ac Mark (Often Found Near Active Regulatory Elements) on 7 cell lines from ENCODE
Layered H3K27Ac
0
Digital DNaseI Hypersensitivity Clusters in 125 cell types from ENCODE
DNase Clusters
Chromatin State Segmentation by HMM from ENCODE/Broad
GM12878 ChromHMM
HMEC ChromHMM
K562 ChromHMM
HepG2 ChromHMM
HUVEC ChromHMM
NHEK ChromHMM
NHLF ChromHMM
HSMM ChromHMM
H1-hESC ChromHMM
Long RNA-seq from ENCODE/Cold Spring Harbor Lab
NHEK nuc pA- A 1
NHEK nuc pA+ A 1
HeG2 cel pA+ A 1
HeG2 cel pA- A 1
HeG2 nuc pA+ A 1
HeG2 cyt pA- A 1
HeG2 cyt pA+ A 1
HeG2 nuc pA- A 1
NHEK cyt pA- A 1
NHEK cyt pA+ A 1
K562 cel pA- A 1
K562 chrm tot A 1
K562 cyt pA- A 1
K562 nuc pA- A 1
K562 nuc pA+ A 2
NHEK cel pA+ A 1
RNA Sub-cellular Localization by Paired-end diTag Sequencing from ENCODE/GIS
GM12 nucl pA+ A 1
HepG2 cytosol polyA+ clone-based RNA PET Alignments Rep 1 from ENCODE/GIS
HepG2 nucleus polyA+ clone-based RNA PET Alignments Rep 1 from ENCODE/GIS

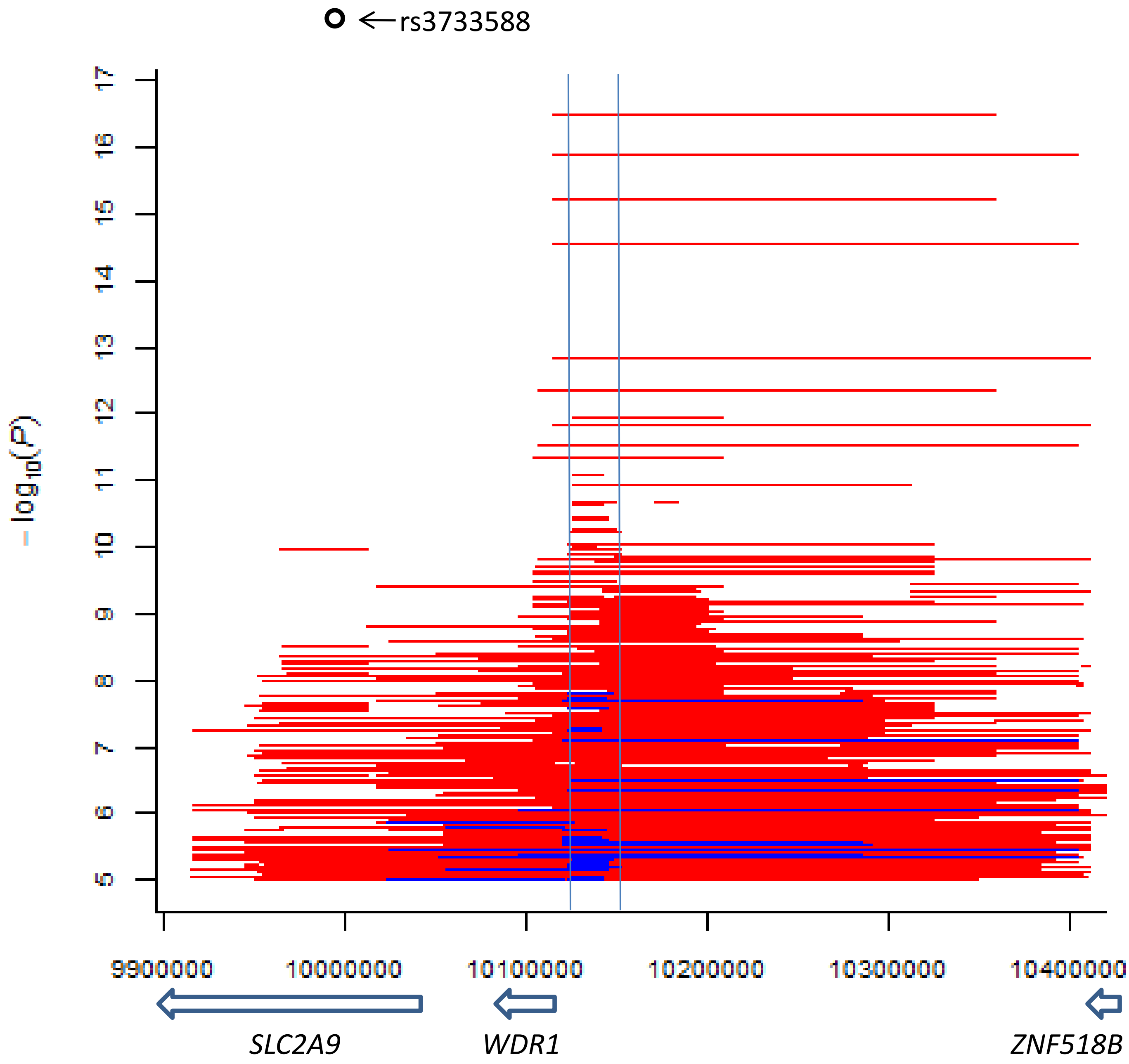

rs3733588
– log10(P)
17
16
15
14
13
12
11
10
9
8
7
6
5
9900000
10000000
10100000
10200000
10300000
10400000
SLC2A9
WDR1
ZNF518B
